# Integrated morphological and physiological plasticity of root for improved seedling growth in *Cunninghamia lanceolata* and *Schima superba* under nitrogen deficiency and different NH_4_^+^-N to NO_3_^−^-N ratio

**DOI:** 10.3389/fpls.2025.1673572

**Published:** 2025-10-27

**Authors:** Yan-Ru Wang, Xiao-Qiang Quan, Xiao-Yu Li, Cong Cheng, Jia-Xiang Yu, Xing-Hao Tang, Peng-Fei Wu, Xiang-Qing Ma, Xiao-Li Yan

**Affiliations:** ^1^ College of Forestry, Fujian Agriculture and Forestry University, Fuzhou, China; ^2^ Engineering Research Center of Chinese Fir, National Forestry and Grassland Administration, Fuzhou, China; ^3^ Fujian Academy of Forestry, Fuzhou, China

**Keywords:** nitrogen deficiency, NH_4_^+^-N to NO_3_^−^-N ratio, root plasticity, *Cunninghamia lanceolata*, *Schima superba*

## Abstract

**Context:**

Root plasticity is an important physiological mechanism for trees to adapt to nitrogen (N) deficiency and the unequal distribution of ammonium nitrogen (NH_4_^+^-N) and nitrate nitrogen (NO_3_^−^-N) for promoting growth. However, the response of root plasticity in *Cunninghamia lanceolata* and *Schima superba* remains unclear.

**Methods:**

A pot experiment with *C*. *lanceolata* and *S. superba* compared N deficiency (0.5 mmol L^-^¹) and normal N (2.0 mmol L^-^¹) under five NH_4_^+^-N to NO_3_^-^-N ratios (10:0, 8:2, 5:5, 2:8, 0:10). Root morphological and physiological traits and seedling growth were investigated.

**Results:**

Under N deficiency, *C*. *lanceolata* showed significantly higher total root surface (TRS) and total root volume (TRV) at higher NO_3_^−^-N concentrations, although the roots were shorter and thicker than normal N. It also showed an increased root tissue density (RTD) while specific root length (SRL) and specific root surface area (SRS) decreased. In contrast, roots of *S. superba* elongated at higher NH_4_^+^-N concentrations, with an increase in TRS, TRV, SRL, and SRS, while RTD decreased. Different N supply treatments significantly affected N-metabolism enzyme activities in the roots of both species. The root biomass of *C*. *lanceolata* and *S. superba* increased compared with normal N supply level, and the relatively high NH_4_^+^-N concentrations favored aboveground growth in both species. Principal component and correlation analysis revealed that there were differences in the response of *C*. *lanceolata* and *S. superba* to different N supply treatments.

**Conclusion:**

Appropriately increasing the application of NH_4_^+^-N under N-deficient conditions promotes the growth of *C*. *lanceolata* and *S. superba*.

## Introduction

Nitrogen (N) is an essential nutrient for maintaining plant growth and regulating resource allocation. The primary inorganic forms of N that plants can directly absorb and utilize are ammonium nitrogen (NH_4_^+^-N) and nitrate nitrogen (NO_3_^−^-N). Still, they comprise a relatively small proportion of the total N in soils. In addition, global climate change has led to rising temperatures in ecosystems and increased atmospheric carbon dioxide (CO_2_) content, disrupting the balance of carbon (C) and N in ecosystems. This disturbing imbalance has indirectly led to an increase in the demand for N by plants (Saxe et al., 2001; Xu et al., 2012). Therefore, N deficiency has become a key factor limiting plant growth compared to N uptake by plants in terrestrial ecosystems (Vitousek and Howarth, 1991; Veresoglou et al., 2012). It has been found that NH_4_^+^-N and NO_3_^−^-N are commonly affected by spatiotemporal variations. Their contents and distributions are highly heterogeneous, especially during the processes of N transformation (Fang et al., 2004; Liu et al., 2017). This unequal distribution of nutrient resources affects the growth and distribution of plant roots, biomass accumulation, and plant interactions (Day et al., 2003; Hodge, 2004). Since soil N deficiency and the unequal distribution of NH_4_^+^-N and NO_3_^−^-N are prevalent in nature, plants have developed a series of regulatory strategies to adapt to these challenges. Over time, these strategies have enhanced their ability to efficiently uptake and utilize both forms of N in nutrient-limited environments. In particular, root plasticity is considered a crucial adaptive strategy that enables plants to capture the limited available N in the soil.

The root system, as an important organ for nutrient absorption and utilization in plants, exhibits functional traits that reflect a series of plastic responses shown by plants in adapting to their environments. The root morphological plasticity of a plant in response to the environment determines the location of the root system in the soil, its ability to expand spatially, and its access to nutrient resources (Duan et al., 2022; Lynch et al., 2024). This response strategy is particularly important in environments that are nutrient-deficient and heterogeneously distributed (Ma et al., 2018; Sun et al., 2023). Numerous studies have shown that morphological traits of the root are significantly influenced by the content and form ratio of N in the soil, which in turn affects N absorption and utilization by plants (Liu, 2010; Kou et al., 2015; Makita et al., 2015; Duan et al., 2022; Liang et al., 2022). For instance, seedlings of *Principis rupprechtii*, *Cotinus coggygria*, and *Pinus koraiensis* adapt to severely nutrient-limited environments primarily by altering root length, root surface area, root tip number, and branching number. Simultaneously, plants adapt to changes in the nutrient environment between the roots by changing the morphological and structural characteristics of the root system and adopting different N-foraging strategies (Xuan et al., 2017; Yan et al., 2020b). Additionally, root physiological plasticity can be frequently adjusted to respond to changes in soil nutrients. Root systems can actively adjust physiological processes, such as the activity of key enzymes involved in N uptake and assimilation, nutrient uptake dynamics, root respiration, and associated gene expression, in order to counteract variations in soil nutrient heterogeneity (Meng, 2016; Liu et al., 2022). N uptake and assimilation, as pivotal physiological processes, are responsible for transporting nutrients to various plant organs (Redinbaugh and Campbell, 1998). Research indicates that N supply levels and varying ratios of NH_4_^+^-N to NO_3_^-^-N can influence N uptake and utilization by enhancing or inhibiting the activity of key enzymes involved in Nitrogen absorption and assimilation (Ma et al., 2022; Vandna et al., 2024). In summary, the root plasticity of the tree is key to elucidating the effective uptake of soil N by the root system and feeds back to determine plant growth and N uptake strategies. However, the effect of different NH_4_^+^-N to NO_3_^–^N ratios on root plasticity under N deficiency has been less investigated.

*Cunninghamia lanceolata* is the predominant fast-growing timber species in southern China, combining important ecological value and economic contribution. However, the problems of pure plantation and continuous multi-generation of *C. lanceolata* have resulted in soil nutrient depletion and stand productivity, which severely limit the sustainable management and development of *C. lanceolata* (Chen et al., 2004; Tian et al., 2011; Liu et al., 2018; Ren et al., 2021). These ecological disturbances have fundamentally affected the processes of N transformation in forest soils, resulting in critical N deficiency that currently constitutes the primary growth-limiting factor for *C. lanceolata* plantations across China (Fang, 1987; Wang et al., 2024). *Schima superba* is an important establishment species of broad-leaved evergreen forests, as well as a major pioneer species for ecological restoration in southern China. It is widely accepted that the decomposition of leaf litter plays a key role in promoting soil nutrient cycling. *S. superba* produces an abundance of leaf litter that decomposes quickly and contains high levels of nutrients. It is more and more often used as a matching tree species to create *C. lanceolata* and *S. superba* mixed forests to solve the practical problems of land force decline and ecological deterioration of *C. lanceolata*. In addition, *C*. *lanceolata* is a shallow-rooted plant, whereas *S. superba* is a deep-rooted plant, and planting the two together can effectively enhance the utilization of nutrients at different levels of the soil (Chen et al., 2021). Currently, large areas of mixed *C. lanceolata* and *S. superba* forests have been established in subtropical regions. These forests provide benefits in terms of increased forest productivity and ecological services. Therefore, it is still necessary to further study the specific responses to N deficiency in a heterogeneous environment of the seedling growth and root morphology of *C. lanceolata* and *S. superba*.

There is an increasing focus on the effects of effective N on the functional traits of root systems in different tree species (Wang et al., 2018; Sell et al., 2022). However, it is also unclear whether different tree species enhance adaptation to nutrient adversity by regulating root system plasticity responses. In addition, previous studies have usually focused separately on root morphological or physiological plasticity, and there has been no unified research on the relationship between the regulatory mechanisms. In this context, we ask the following research questions: (1) How do N deficiency and different NH_4_^+^-N to NO_3_^−^-N ratios affect the root morphology, physiology, and seedling growth in *C. lanceolata* and *S. superba* (2) How do the two species adapt to N deficiency and different NH_4_^+^-N to NO_3_^−^-N ratios by regulating root plasticity of their root systems, respectively, to promote seedling growth? (3) Under the same conditions of increasing soil N deficit and heterogeneous distribution environments, do the two species exhibit differing response strategies in root morphology and physiology? To address these questions, we investigated the effect of N deficiency and different NH_4_^+^-N to NO_3_^−^-N ratios on root morphological and physiological traits and seedling growth of *C. lanceolata* and *S. superba*. The aim was to investigate the mechanisms of N uptake in the roots of the two species under conditions of N deficiency and to provide a theoretical basis for the suitability of mixed stands of *C. lanceolata* and *S. superba* to improve the efficiency of limited N.

## Materials and methods

### Plant material and experimental conditions

The test materials for our investigation were *C. lanceolata* and *S. superba*, the predominant conifer and broadleaf species in subtropical regions, respectively. *C. lanceolata* is an economically important timber species for afforestation in China. It has the advantages of high yield, excellent timber quality, and significant economic benefits (Yan and Ma, 2021). *S. superba* is a significant tree species in the evergreen broadleaf forests. It also serves as a crucial pioneer species for ecological restoration owing to its excellent material and adaptability (Chen and Hua, 2017). These two species are often planted together in mixed plantations to improve the structure of stands and their ecological functions and to promote the natural regeneration of forests in southern China.

In April 2022, one-year-old seedlings of *C. lanceolata* and *S. superba* were selected for this experiment. These seedlings were free from pests and diseases and had similar root systems. They were cultivated in a well-ventilated and permeable greenhouse. All seedlings were purchased from the state-owned Zhangping Wuyi Forest Farm, Fujian, China.

### Experimental design and culture of the seedlings

A pot experiment was designed to investigate the effects of N deficiency and different NH_4_^+^-N to NO_3_^−^-N ratios on the seedling growth, root morphology, and root physiological traits of *C*. *lanceolata* and *S*. *superba*. Seedlings were grown in plastic and columniform pots (22.5 cm in diameter and 24.7 cm in depth). All the pots were placed in the greenhouse in a completely randomized manner. The pots were filled with pretreated sand that was continuously washed with distilled water until the N content approached zero, creating an environment without any additional N sources (Wu et al., 2011). After thorough washing, the sand was steam-pasteurized at a temperature of 120°C for 30 minutes to minimize the potential risk of bacterial inoculation in the root. Each pot was planted with a single seedling positioned in the center of the plastic pot and supplied with a different concentration of N solution.

The study set up two N supply levels: 0.5 mmol L^-1^ and 2.0 mmol L^-1^, which represent N deficiency and normal N supply level, respectively. The normal N supply level is based on the results of previous studies (Zhang et al., 2006; Meng, 2016). At the two N supply levels, different concentrations of NH_4_^+^-N and NO_3_^−^-N in the nutrient solution resulted in five different NH_4_^+^-N to NO_3_^−^-N ratios (NH_4_^+^-N to NO_3_^−^-N ratios of 10:0, 8:2, 5:5, 2:8, and 0:10). Experimental controls included normal N supply level and homogenous N supply (NH_4_^+^-N to NO_3_^−^-N ratio of 5:5). There was a total of ten treatments, and each treatment had six replications. Each pot received a modified Hoagland solution that contained the necessary macronutrients (Hoagland formula) and micronutrients (Amon formula) to meet the requirements for the seedling growth of *C*. *lanceolata* and *S*. *superba*. Furthermore, the nutritional solution contains 7 μmol L^-1^ nitrification inhibitor (C_2_H_4_N_4_) to prevent the NH_4_^+^-N from converting into NO_3_^−^-N (Sun et al., 2015; Liang et al., 2022). NH_4_^+^-N was supplied in the form of (NH_4_)_2_SO_4_, while NO_3_^−^-N was supplied in the form of NaNO_3_. The pH of the nutritional solution was adjusted to 5.50 ± 0.05 using 2.0 mol L^-1^ NaOH and HCl solutions. In each treatment, NaCl was used to compensate for the difference in Na^+^ concentration. During the experimental period, each treatment was watered with an equal amount of pure water (50 mL to 100 mL) poured once every 2 days, and 50 mL of the nutrient solution was poured every 5 days. The experiment lasted for 180 days.

### Plant harvesting and data measuring

In mid-October 2022, the final height and ground diameter of each one-and-a-half-year-old seedling were measured and recorded. The height was measured using a 50 cm steel ruler, while the ground diameter was measured using a vernier caliper. The seedlings were carefully uprooted in two batches at a time. To minimize damage to the root system, gently shake the pot to loosen the sandy substrate when pulling up the seedlings. For the first batch, three replicates were randomly selected for harvesting, and approximately 1 to 1.5 g of fresh fine roots were clipped and placed in sealed bags to measure N-metabolism enzyme activities. The key enzyme activities of the N metabolism that were measured included glutamine synthetase activity (GS), glutamate synthase activity (GOGAT), nitrate reductase activity (NR), nitrite reductase activity (NiRs) and glutamate dehydrogenase activity (GDH). The measurement process was conducted by Allwegene Tech. Co., Ltd. As destructive sampling was conducted on all target seedlings to measure root N metabolism enzyme activities, the remaining three replicates were used for the measurement of root morphology to ensure the accuracy of root morphology and biomass. The seedlings were divided into roots, stems, and leaves. To ensure the integrity and cleanliness of the root systems, the roots of all seedlings are gently washed with a stream of water, and then carefully dried using absorbent paper. The roots were placed in a plexiglass tray (18 cm × 26 cm) without overlapping, using forceps to arrange them. All roots were scanned using an Epson Expression 12000XL scanner (Seiko Epson Corporation, Suwa, Nagano, Japan). The root morphological characteristic parameters, including the total root length (TRL, cm·tree^-1^), total root surface area (TRS, cm^2^·tree^-1^), total root volume (TRV, cm^3^·tree^-1^), and the average root diameter (ARD, mm·tree^-1^), were determined using a WinRhizo root analysis system (Pro2017a, Regent Instruments Inc., Quebec, Canada). After scanning, the stems, leaves, and roots of the harvested plants were oven-dried at 65 °C until a constant weight was achieved, and the biomass was measured with an accuracy of 0.001 g.

The specific root length (SRL, g·cm^-1^) was calculated as the TRL divided by the total root biomass (TRB, g·tree^-1^). The specific root surface area (SRS, g·cm^-2^) was calculated by dividing the TRS by the TRB. The root tissue density (RTD, g·cm^-3^) was calculated as the ratio of TRB to TRV. The height increment (Δ*H*, cm) and ground diameter increment (Δ*GD*, mm) were calculated as the treated seedling height and ground diameter minus the initial seedling height and ground diameter, respectively. The aboveground biomass (TAB, g·tree^-1^) of an individual plant was calculated as the sum of the dry weights of leaves and stems. The root-to-shoot ratio (RSR) was calculated as the TRB divided by the TAB. The total seedling biomass (TB, g·tree^-1^) of an individual plant was calculated as the sum of TRB and TAB.

### Statistical analysis

To assess the root responses and growth, we calculated the mean values and standard errors for seedling growth, as well as root morphological and physiological traits in each pot across the two N supply levels and five different NH_4_^+^-N to NO_3_^−^-N ratios. All statistical analysis was conducted using the SPSS 25.0 software (SPSS Inc., Chicago, Illinois, USA). Three-way ANOVA was performed to analyze the effects of different N supply levels, different NH_4_^+^-N to NO_3_^−^-N ratios, and tree species on seedling growth and root morphological and physiological traits. One-way ANOVA was performed to compare differences and evaluate the effect of NH_4_^+^-N to NO_3_^−^-N ratios on the TRL, TRS, TRV, ARD, SRL, SRV, RTD, Δ*H*, Δ*GD*, TRB, TAB, TB, RSR, GS, GOGAT, NR, NiRs, and GDH at the same N supply level. Differences between N supply levels at the same NH_4_^+^-N to NO_3_^−^-N ratio and between the two species under the same N treatments were tested using an independent sample t-test. Means showing significant differences were further compared using the Duncan significance test (the significance level was set at α = 0.05). A principal component analysis (PCA) was conducted on the changes and response strategies in suites of root traits and seedling growth of two species under different N treatments. Relationships among root morphological traits, root physiological traits, and seedling growth were assessed using Pearson correlations, and their results were visualized in a heatmap. Histograms, PCA biplot, and heatmap were plotted using Origin 2021 software (Origin Lab, Northampton, USA).

## Results

### Effects of N supply level, NH_4_^+^-N to NO_3_^−^-N ratio, tree species, and their interactions on root morphological and physiological traits and seedling growth

The N supply level had no significant effect on the ARD, SRL, SRS, and RTD. However, it significantly affected the TRL, TRS, TRV, seedling growth (Δ*H*, Δ*GD*, TRB, TAB, TB, and RSR), and root physiological traits (GS, GOGAT, NR, NiRs, and GDH). The interaction between N supply level and tree species significantly affected most of the parameters. NH_4_^+^-N to NO_3_^–^N ratio, tree species, and their interaction significantly affected most of the parameters. However, the interaction between the N supply level and NH_4_^+^-N to NO_3_^–^N ratio, as well as the interaction among N supply level, NH_4_^+^-N to NO_3_^–^N ratio, and tree species, did not reach significant levels for most of the parameters except for root physiological traits (**Table 1**).

**Table 1:** The *p*-values of three-way ANOVA for the effects of N supply level (N), NH_4_^+^-N to NO_3_^–^N ratio (R), tree species (T), and their interactions on the root morphological and physiological traits and the seedling growth.

Factor	*P*-value and significance level						
	N supply level (N)	NH_4_^+^-N to NO_3_^–^N ratio (R)	Tree species (T)	N×R	N×T	R×T	N×R×T
Total root length (TRL)	<0.001***	0.021*	<0.001***	0.022*	0.001**	<0.001***	0.045*
Total root surface area (TRS)	0.012*	0.298NS	<0.001***	0.969NS	0.084NS	<0.001***	0.517NS
Total root volume (TRV)	<0.001***	<0.001***	<0.001***	0.365NS	<0.001***	<0.001***	0.100NS
Average root diameter (ARD)	0.109NS	0.001**	<0.001***	0.219 NS	0.013*	<0.001***	0.022*
Specific root length (SRL)	0.224NS	<0.001***	<0.001***	0.043*	<0.001***	<0.001***	0.317NS
Specific root surface area (SRS)	0.283NS	<0.001***	0.105NS	0.909NS	0.003**	0.032*	0.792NS
Root tissue density (RTD)	0.751NS	<0.001***	<0.001***	0.076NS	<0.001***	0.003**	0.467NS
Glutamine synthetase activity (GS)	<0.001***	<0.001***	<0.001***	<0.001***	<0.001***	<0.001***	<0.001***
Glutamate synthase activity (GOGAT)	<0.001***	<0.001***	<0.001***	<0.001***	<0.001***	<0.001***	<0.001***
Nitrate reductase activity (NR)	<0.001***	<0.001***	0.009**	<0.001***	<0.001***	<0.001***	<0.001***
Nitrite reductase activity (NiRs)	<0.001***	<0.001***	<0.001***	<0.001***	<0.001***	<0.001***	<0.001***
Glutamate dehydrogenase activity (GDH)	<0.001***	<0.001***	<0.001***	<0.001***	<0.001***	<0.001***	<0.001***
Ground diameter increment (Δ*GD*)	<0.001***	<0.001***	<0.001***	<0.001***	0.928NS	<0.001***	<0.001***
Height increment (Δ*H*)	<0.001***	0.088NS	<0.001***	0.584NS	0.621NS	0.111NS	0.136NS
Total root biomass (TRB)	<0.001***	<0.001***	<0.001***	0.900NS	0.017*	<0.001***	0.296NS
Total aboveground biomass (TAB)	<0.001***	<0.001***	<0.001***	0.602NS	0.009**	<0.001***	0.107NS
Total biomass (TB)	0.001**	0.005**	<0.001***	0.520NS	0.038*	0.015*	0.054NS
Root-to-shoot ratio (RSR)	<0.001***	<0.001***	<0.001***	0.494NS	0.030*	<0.001***	0.183NS

Significance of analysis of variance factor: NS, not significant, *, *p* < 0.05, **, *p* < 0.01, ***, *p* < 0.001

### Effects of Different N Supply Levels and NH_4_^+^-N to NO_3_^−^-N Ratios on Root Morphological Traits.

**Figure 1:**
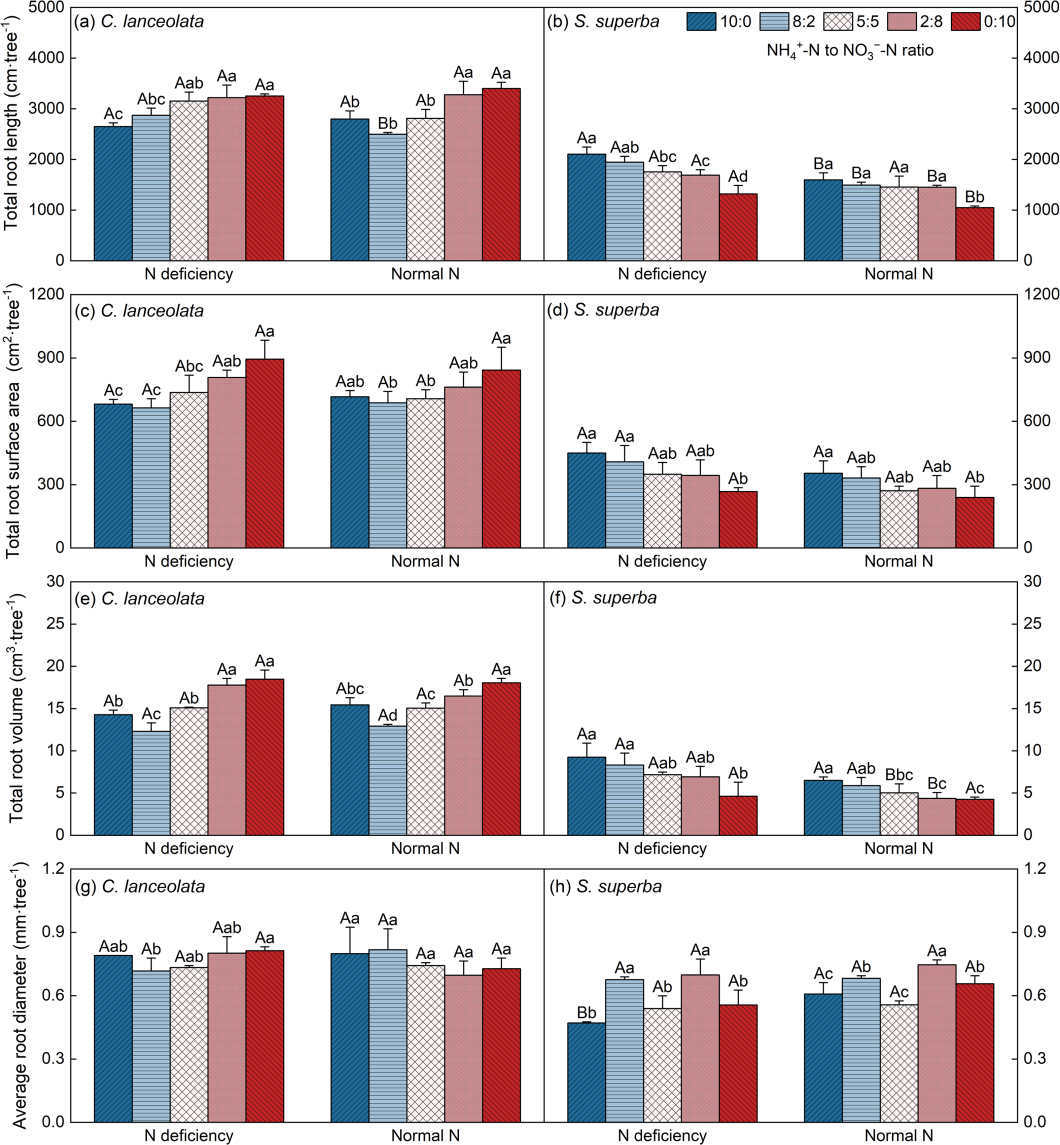
Effects of different N supply levels and NH_4_^+^-N to NO_3_^–^N ratios on the TRL **(a, b)**, TRS **(c, d)**, TRV **(e, f)**, and ARD **(g, h)** of *C. lanceolata* and *S. superba*. Values are expressed as mean and standard deviation (n=3). Different capital letters indicate the significant difference between the two N supply levels at the same NH_4_^+^-N to NO_3_^–^N ratio, and different lowercase letters indicate the significant difference between the five NH_4_^+^-N to NO_3_^–^N ratios at the same N supply level (*P* < 0.05).

**Figure 2:**
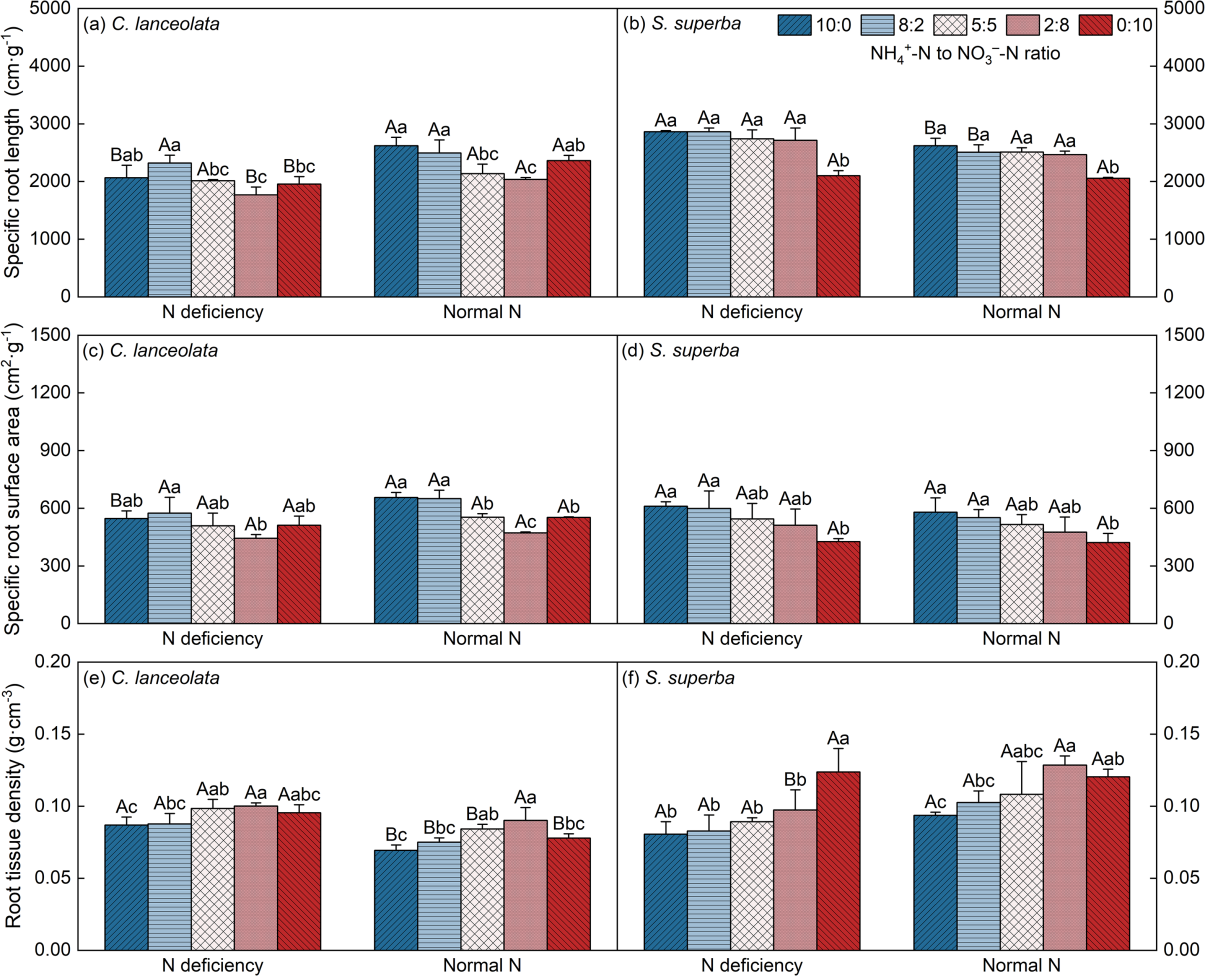
Effects of different N supply levels and NH_4_^+^-N to NO_3_^–^N ratios on the SRL **(a, b)**, SRS **(c, d)**, and RTD **(e, f)** of *C. lanceolata* and *S. superba*. Values are expressed as mean and standard deviation (n=3). Different capital letters indicate the significant difference between the two N supply levels at the same NH_4_^+^-N to NO_3_^–^N ratio, and different lowercase letters indicate the significant difference between the five NH_4_^+^-N to NO_3_^–^N ratios at the same N supply level (*P* < 0.05).

Under N deficiency, the TRL of *C. lanceolata* was significantly higher than that under normal N supply only at an NH_4_^+^-N to NO_3_^−^-N ratio of 8:2 (**Figure 1a**). Difference in TRS, TRV, and ARD of *C. lanceolata* between the two N supply levels were not significant at all five NH_4_^+^-N to NO_3_^−^-N ratios (**Figures 1c, e, g**). The SRL and SRS of *C. lanceolata* were lower at all five NH_4_^+^-N to NO_3_^−^-N ratios under N deficiency, with significant differences only at NH_4_^+^-N to NO_3_^−^-N ratio of 10:0 (**Figures 2a, c**). However, the RTD of *C. lanceolata* was higher under N deficiency, showing a significant increase at NH_4_^+^-N to NO_3_^−^-N ratios of 10:0, 8:2, 5:5, and 0:10 (**Figure 2e**).

Under five NH_4_^+^-N to NO_3_^−^-N ratios, the TRL, TRS, and TRV of *S*. *superba* under N deficiency were all higher than those under normal N supply, with maximum values occurring at an NH_4_^+^-N to NO_3_^−^-N ratio of 10:0 (**Figures 1b, d, f**). However, the ARD of *S*. *superba* decreased under all five NH_4_^+^-N to NO_3_^−^-N ratios compared to normal N supply, with the smallest diameter observed at NH_4_^+^-N to NO_3_^−^-N of 2:8 (Figure1h).

Compared with a normal N supply, *C. lanceolata* treated with N deficiency exhibited lower SRL and SRS, alongside higher RTD (**Figures 2a, c, e**). Conversely, *S*. *superba* demonstrated a higher SRL and SRS, coupled with a lower RTD (**Figures 2b, d, f**). Under N deficiency, both *C. lanceolata* and *S*. *superba* exhibited greater SRL and SRS, alongside reduced RTD, when subjected to relatively high NH_4_^+^-N concentrations.

For all NH_4_^+^-N to NO_3_^−^-N ratios and N supply levels, TRL, TRS, TRV and ARD were higher in *C. lanceolata* than in *S*. *superba*. Under normal supply level, significant differences in SRL occurred at ratios of 5:5, 2:8, and 0:10, and in SRS at 8:2 and 0:10. RTD was similar between species at NH_4_^+^-N to NO_3_^−^-N ratio of 5:5. Under N deficiency, the SRL of *C. lanceolata* was comparable to that of *S*. *superba* at NH_4_^+^-N to NO_3_^−^-N of 0:10 ratio. Its SRS and RTD showed no significant differences at NH_4_^+^-N to NO_3_^−^-N of 10:0, 8:2, 5:5, and 2:8 NH_4_^+^ to NO_3_^-^ ratios (**Table 2**).

**Table 2:** Differences in the root morphological and physiological traits and the seedling growth between *C. lanceolata* and *S. superba* under N deficiency and the different NH_4_^+^-N to NO_3_^–^N ratios.

Index	*P*-value and significance level									
	N deficiency					Normal N				
	10:0	8:2	5:5	2:8	0:10	10:0	8:2	5:5	2:8	0:10
TRL	0.004**	<0.001***	<0.001***	<0.001***	<0.001***	<0.001***	<0.001***	<0.001***	<0.001***	<0.001***
TRS	0.002**	0.008**	0.002**	<0.001***	<0.001***	<0.001***	<0.001***	<0.001***	<0.001***	<0.001***
TRV	0.008**	0.016*	<0.001***	<0.001***	<0.001***	<0.001***	<0.001***	<0.001***	<0.001***	<0.001***
ARD	<0.001***	0.310NS	0.005**	0.174NS	0.004**	0.075NS	0.077NS	<0.001***	0.310NS	0.130NS
SRL	0.003**	0.003**	<0.001***	0.003**	0.181NS	0.997NS	0.924NS	0.022*	<0.001***	0.004**
SRS	0.073NS	0.751NS	0.581NS	0.247NS	0.041*	0.166NS	0.046*	0.291NS	0.928NS	0.009**
RTD	0.352NS	0.560NS	0.083NS	0.753NS	0.046*	<0.001***	0.005**	0.146NS	0.004**	<0.001***
GS	<0.001***	<0.001***	<0.001***	<0.001***	<0.001***	<0.001***	<0.001***	<0.001***	<0.001***	<0.001***
GOGAT	<0.001***	<0.001***	<0.001***	<0.001***	<0.001***	<0.001***	<0.001***	<0.001***	<0.001***	<0.001***
NR	<0.001***	<0.001***	<0.001***	<0.001***	<0.001***	<0.001***	<0.001***	<0.001***	<0.001***	<0.001***
NiRS	<0.001***	<0.001***	0.016*	<0.001***	<0.001***	<0.001***	<0.001***	0.2985NS	<0.001***	<0.001***
GDH	<0.001***	<0.001***	<0.001***	<0.001***	<0.001***	<0.001***	0.075NS	<0.001***	<0.001***	<0.001***
Δ*GD*	0.004**	<0.001***	<0.001***	0.013*	0.003**	<0.001***	<0.001***	<0.001***	0.024*	0.013*
Δ*H*	0.365NS	0.004**	0.113NS	0.617NS	0.080NS	0.006**	0.005**	0.754NS	0.494NS	0.173NS
TRB	0.005**	<0.001***	<0.001***	<0.001***	<0.001***	0.003**	0.040*	0.014*	0.002**	<0.001***
TAB	0.007**	0.002**	<0.001***	0.223NS	0.082NS	0.031*	0.009**	0.062NS	0.023*	0.003**
TB	0.003**	<0.001***	<0.001***	<0.001***	0.005**	0.003**	0.010*	0.012*	<0.001***	<0.001***
RSR	0.952NS	0.276NS	0.020*	0.022*	<0.001***	0.015*	0.117NS	0.145NS	<0.001***	0.005**

Significance of analysis of variance factor: NS, not significant, *, *p* < 0.05, **, *p* < 0.01, ***, *p* < 0.001.

### Effects of different N supply levels and NH_4_^+^-N to NO_3_^−^-N ratios on root physiological traits

**Figure 3:**
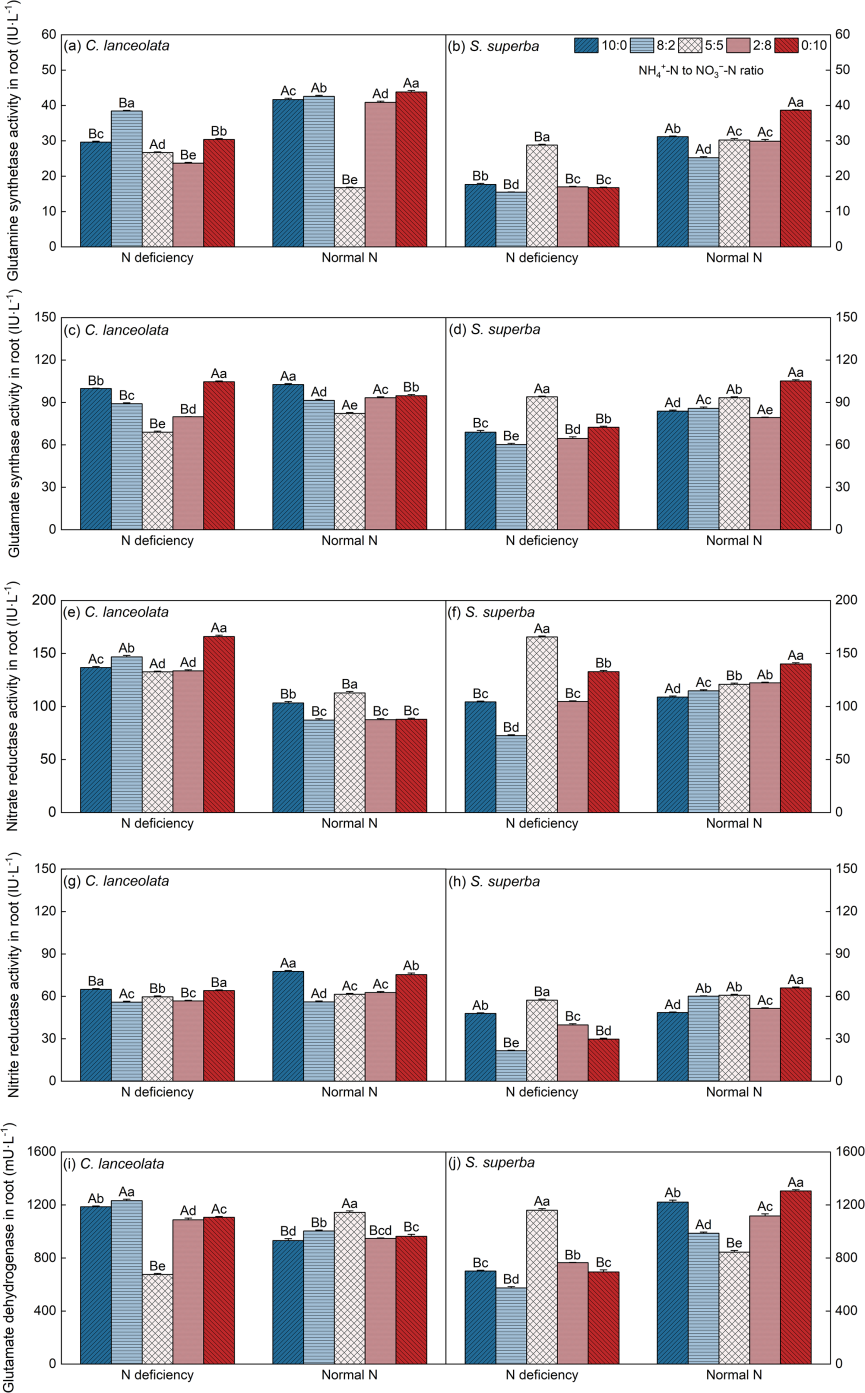
Effects of different N supply levels and NH_4_^+^-N to NO_3_^–^N ratios on the GS **(a, b)**, GOGAT **(c, d)**, NR **(e, f)**, NiRs **(g, h)**, and GDH **(i, j)** of *C. lanceolata* and *S. superba*. Values are expressed as mean and standard deviation (n=3). Different capital letters indicate the significant difference between the two N supply levels at the same NH_4_^+^-N to NO_3_^–^N ratio, and different lowercase letters indicate the significant difference between the five NH_4_^+^-N to NO_3_^–^N ratios at the same N supply level (*P* < 0.05).

From the results of **Figure 3**, it is clear that the activities of GS, GOGAT, and NiRs in roots of *C. lanceolata* generally showed a downward trend under N deficiency at most NH_4_^+^-N to NO_3_^−^-N ratios compared to normal N supply level, while NR and GDH showed the opposite trend. The difference between the two N supply levels was significant. Under N deficiency, *C. lanceolata* did not display an obvious pattern across different NH_4_^+^-N to NO_3_^−^-N ratios. In contrast, for *S. superba*, the activities of key N metabolism enzymes were significantly higher at an NH_4_^+^-N to NO_3_^−^-N ratio of 5:5 compared to normal N supply. However, under N deficiency, the activities of GS, GOGAT, NR, NiRs, and GDH in the roots of *S. superba* generally decreased across the remaining NH_4_^+^-N to NO_3_^−^-N ratios. The differences between the two N supply levels were significant at most NH_4_^+^-N to NO_3_^−^-N ratios.

Under N deficiency treatment, the activities of GS, GOGAT, NR, and GDH in the roots of *C. lanceolata* at the NH_4_^+^-N to NO_3_^−^-N ratio of 5:5 lower than those of *S*. *superba*, with differences being highly significant. In contrast, at the other four NH_4_^+^-N to NO_3_^−^-N ratios, the activities of these enzymes were higher in *C. lanceolata*. Additionally, the activity of NiRs in the roots of *C. lanceolata* was significantly higher than that of *S*. *superba* across all five NH_4_^+^-N to NO_3_^−^-N ratios (**Table 2**).

### Effects of different N supply levels and NH_4_^+^-N to NO_3_^−^-N ratios on seedling growth and biomass

**Figure 4:**
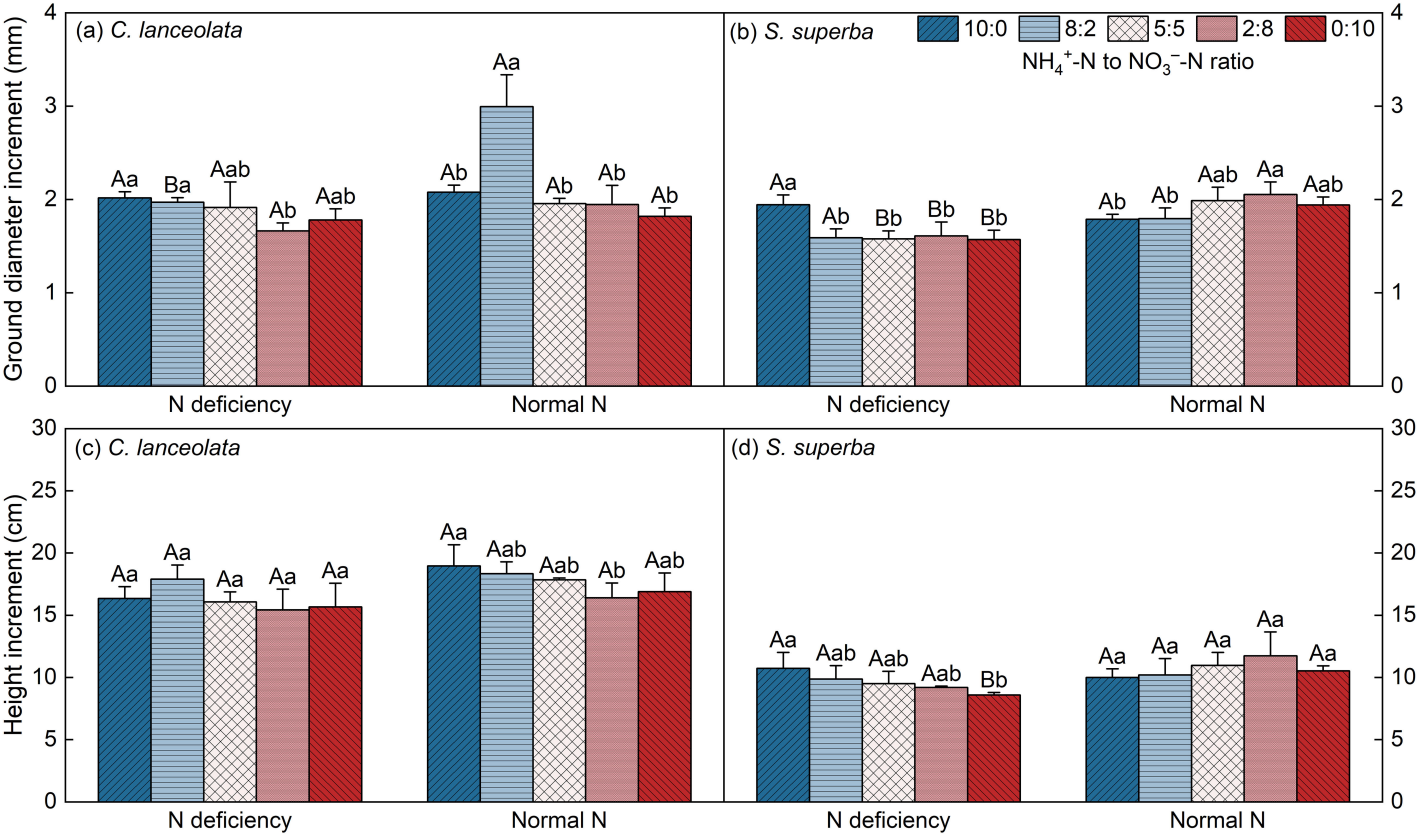
Effects of different N supply levels and NH_4_^+^-N to NO_3_^–^-N ratios on the ground diameter increments **(a, b)** and the height increments **(c, d)** of *C. lanceolata* and *S. superba*, respectively. Values are expressed as mean and standard deviation (n=3). Different capital letters indicate the significant difference between the two N supply levels at the same NH_4_^+^-N to NO_3_^–^-N ratio, and different lowercase letters indicate the significant difference between the five NH_4_^+^-N to NO_3_^–^-N ratios at the same N supply level (*P*<0.05).

The Δ*H* and Δ*GD* of *C. lanceolata* under N deficiency were lower than that under normal N supply at all five NH_4_^+^-N to NO_3_^–^N ratios, and showed a preference for high NH_4_^+^-N concentrations > homogenous N supply > high NO_3_^−^-N concentrations under both N supply levels (**Figures 4a, c**). The Δ*H* and Δ*GD* of *S*. *superba* under N deficiency were decreased at most NH_4_^+^-N to NO_3_^−^-N ratios. Under normal N supply, the Δ*H* and Δ*GD* of *S*. *superba* showed a preference for high NO_3_^−^-N concentrations and homogenous N supply>high NH_4_^+^-N concentrations, while under N deficiency, both of them showed high NH_4_^+^-N concentrations>homogenous N supply>high NO_3_^−^-N concentrations (**Figures 4b, d**).

Under N deficiency, the TRB and RSR of *C. lanceolata* were higher than that of normal N supply at all five NH_4_^+^-N to NO_3_^–^N ratios and followed the same pattern of 2:8 > 0:10 > 5:5 > 10:0 > 8:2 under the two N supply levels. The TRB and RSR of *S*. *superba* were higher than that under normal N supply at most NH_4_^+^-N to NO_3_^−^-N ratios all four NH_4_^+^-N to NO_3_^–^N ratios except for the NH_4_^+^-N to NO_3_^−^-N ratio of 2:8, and was larger at relatively high NH_4_^+^-N concentrations in TRB. The RSR of *S*. *superba* under N deficiency was as follows: homogenous N supply > high NH_4_^+^-N concentrations > high NO_3_^−^-N concentrations. However, the TAB and TB of *C. lanceolata* and *S*. *superba* were decreased under N deficiency at most NH_4_^+^-N to NO_3_^−^-N ratios, and both accumulated more at relatively high NH_4_^+^-N concentrations (**Table 3**).

**Table 3:** Effects of different N supply levels and NH_4_^+^-N to NO_3_^–^N ratios on total root biomass (TRB), total aboveground biomass (TAB), total seedling biomass (TB), and root-to-shoot ratio (RSR).

Tree species	N supply level	NH_4_^+^-N to NO_3_^–^N ratio	TRB (g)	TAB (g)	TB (g)	RSR
*C*. *lanceolata*	N deficiency	10:0	1.294 ± 0.167Acd	4.001 ± 0.590Aa	5.295 ± 0.597Aa	0.328 ± 0.070Abc
		8:2	1.164 ± 0.091Ad	3.818 ± 0.384Aab	4.982 ± 0.456Aa	0.306 ± 0.021Bc
		5:5	1.581 ± 0.183Abc	3.417 ± 0.142Aabc	4.998 ± 0.315Aa	0.462 ± 0.038Abc
		2:8	2.110 ± 0.290Aa	2.613 ± 0.620Ac	4.723 ± 0.404Aa	0.848 ± 0.267Aa
		0:10	1.667 ± 0.090Ab	2.949 ± 0.712Abc	4.616 ± 0.626Aa	0.591 ± 0.163Aab
	Normal N	10:0	1.071 ± 0.119Ab	5.761 ± 1.201Aa	6.832 ± 1.116Aa	0.193 ± 0.051Ab
		8:2	1.010 ± 0.233Ab	5.278 ± 1.038Aa	6.288 ± 1.200Aab	0.192 ± 0.036Ab
		5:5	1.323 ± 0.177Aab	4.106 ± 1.055Aab	5.429 ± 0.921Aab	0.342 ± 0.126Aab
		2:8	1.635 ± 0.234Aa	3.351 ± 0.384Ab	4.986 ± 0.168Ab	0.497 ± 0.123Aa
		0:10	1.472 ± 0.209Aa	3.265 ± 0.291Ab	4.737 ± 0.399Ab	0.452 ± 0.063Aa
*S*. *superba*	N deficiency	10:0	0.735 ± 0.055Aa	2.222 ± 0.148Aa	2.957 ± 0.181Aa	0.331 ± 0.023Aab
		8:2	0.680 ± 0.026Aab	2.087 ± 0.118Bab	2.766 ± 0.128Aab	0.326 ± 0.018Aab
		5:5	0.640 ± 0.010Abc	1.823 ± 0.192Bb	2.463 ± 0.201Bc	0.354 ± 0.033Aa
		2:8	0.573 ± 0.062Ac	2.078 ± 0.169Bab	2.651 ± 0.108Bbc	0.278 ± 0.053Ab
		0:10	0.628 ± 0.060Abc	1.982 ± 0.141Aab	2.610 ± 0.111Abc	0.319 ± 0.047Aab
	Normal N	10:0	0.609 ± 0.023Ba	1.896 ± 0.050Bb	2.505 ± 0.029Bb	0.322 ± 0.020Aa
		8:2	0.598 ± 0.053Aa	2.446 ± 0.132Aa	3.044 ± 0.130Aa	0.245 ± 0.029Bb
		5:5	0.527 ± 0.031Bb	2.519 ± 0.157Aa	3.046 ± 0.155Aa	0.210 ± 0.019Bb
		2:8	0.590 ± 0.030Aa	2.481 ± 0.175Aa	3.071 ± 0.205Aa	0.238 ± 0.005Ab
		0:10	0.511 ± 0.012Bb	2.126 ± 0.100Ab	2.636 ± 0.090Ab	0.241 ± 0.016Ab

The Δ*GD* of *C. lanceolata* was significantly higher under N deficiency than that of *S*. *superba* at NH_4_^+^-N to NO_3_^−^-N ratio of 8:2, and was significantly higher than that of *S*. *superba* at NH_4_^+^-N to NO_3_^−^-N ratios of 10:0 and 8:2 under normal N supply. At all five NH_4_^+^-N to NO_3_^−^-N ratios, the Δ*H*, TRB, TAB, and TB of *C. lanceolata* were significantly higher than that of *S*. *superba* under the two N supply levels. The RSR of *C. lanceolata* was lower than that of *S*. *superba* at NH_4_^+^-N to NO_3_^−^-N ratios of 10:0 and 8:2, and higher at NH_4_^+^-N to NO_3_^−^-N ratios of 5:5, 2:8, and 0:10 under both N supply levels (**Table 2**).

### Principal component analysis of root morphological and physiological traits and seedling growth of *C. lanceolata* and *S. superba* under N deficiency and NH_4_^+^-N to NO_3_^−^-N ratio

**Figure 5:**
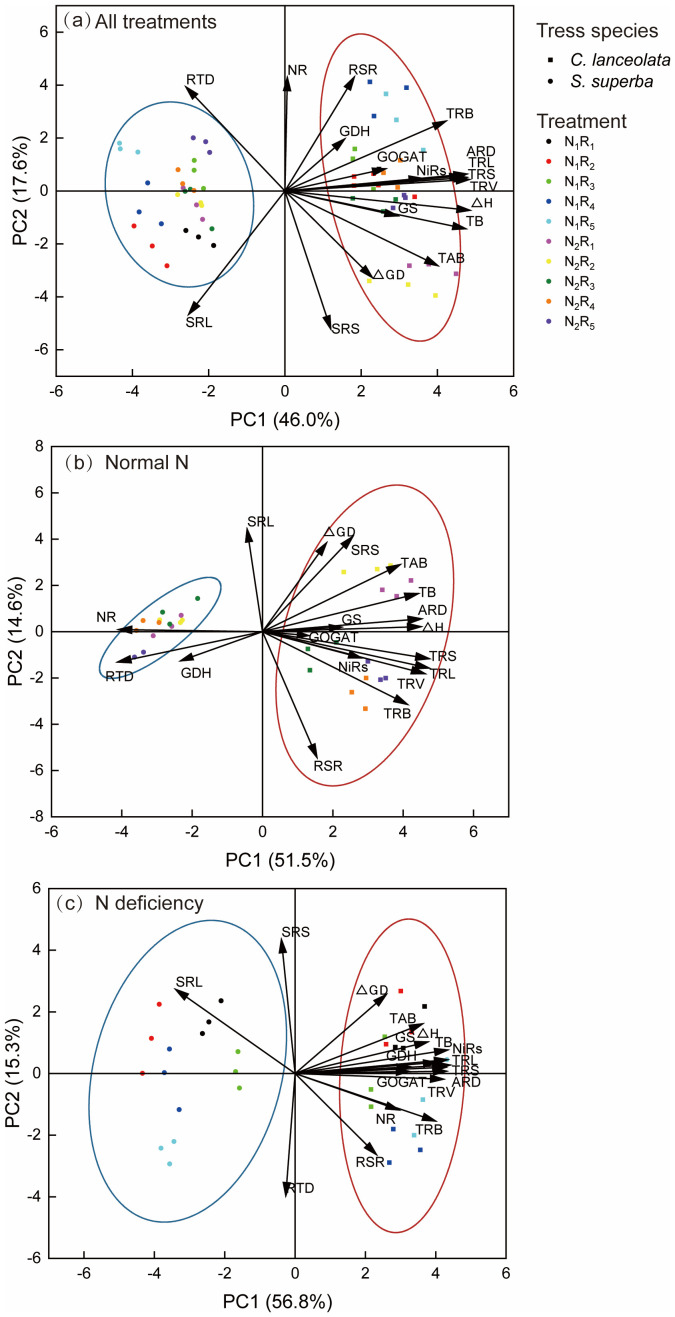
The principal component biplot for the root morphological and physiological traits and the seedling growth of *C. lanceolata* and *S. superba* under N deficiency and NH_4_^+^-N to NO_3_^–^-N ratio was analyzed. Different symbols indicate different species. Different colors indicate different N treatments. **(a)** All treatments, **(b)** Normal N, **(c)** N deficiency. N_1_ represents N deficiency, N_2_ represents normal N, and R_1_, R_2_, R_3_, R_4_ and R_5_ represent NH_4_^+^-N to NO_3_^−^-N ratio of 10:0, 8:2, 5:5, 2:8 and 0:10, respectively.

Overall, the contribution of PC1 was 46%, and the contribution of PC2 was 17.6%. *C. lanceolata* was mainly distributed on the positive half-axis of PC1, and *S. superba* was mainly distributed on the negative half-axis of PC1. It shows that the two species are significantly different (**Figure 5a**). Under normal N supply, the contribution of PC1 was 51.1%, and the contribution of PC2 was 14.6%. *S. superba* showed some overlap under different NH_4_^+^-N to NO_3_^–^N ratios, indicating that *S. superba* varied less under different NH_4_^+^-N to NO_3_^–^N ratios, whereas *C. lanceolata* varied more (**Figure 5b**). Under N deficiency, the contribution of PC1 was 56.8%, and the contribution of PC2 was 15.3%. The differential responses of *C. lanceolata* to varying NH_4_^+^-N to NO_3_^-^-N ratios remained relatively consistent compared with the normal N supply level, whereas those of *S. superba* exhibited progressively greater differential responses Furthermore, most of the variables of root morphology, physiology, and seedling growth of *C. lanceolata* under N deficiency contributed more to PC1, especially the GS and GOGAT. The contribution of SRL to *S. superba* increased (**Figures 5b, c**).

### Correlation among root morphology, physiological traits, and growth of *C. lanceolata* and *S. superba* under N deficiency and NH_4_^+^-N to NO_3_^−^-N ratio

**Figure 6:**
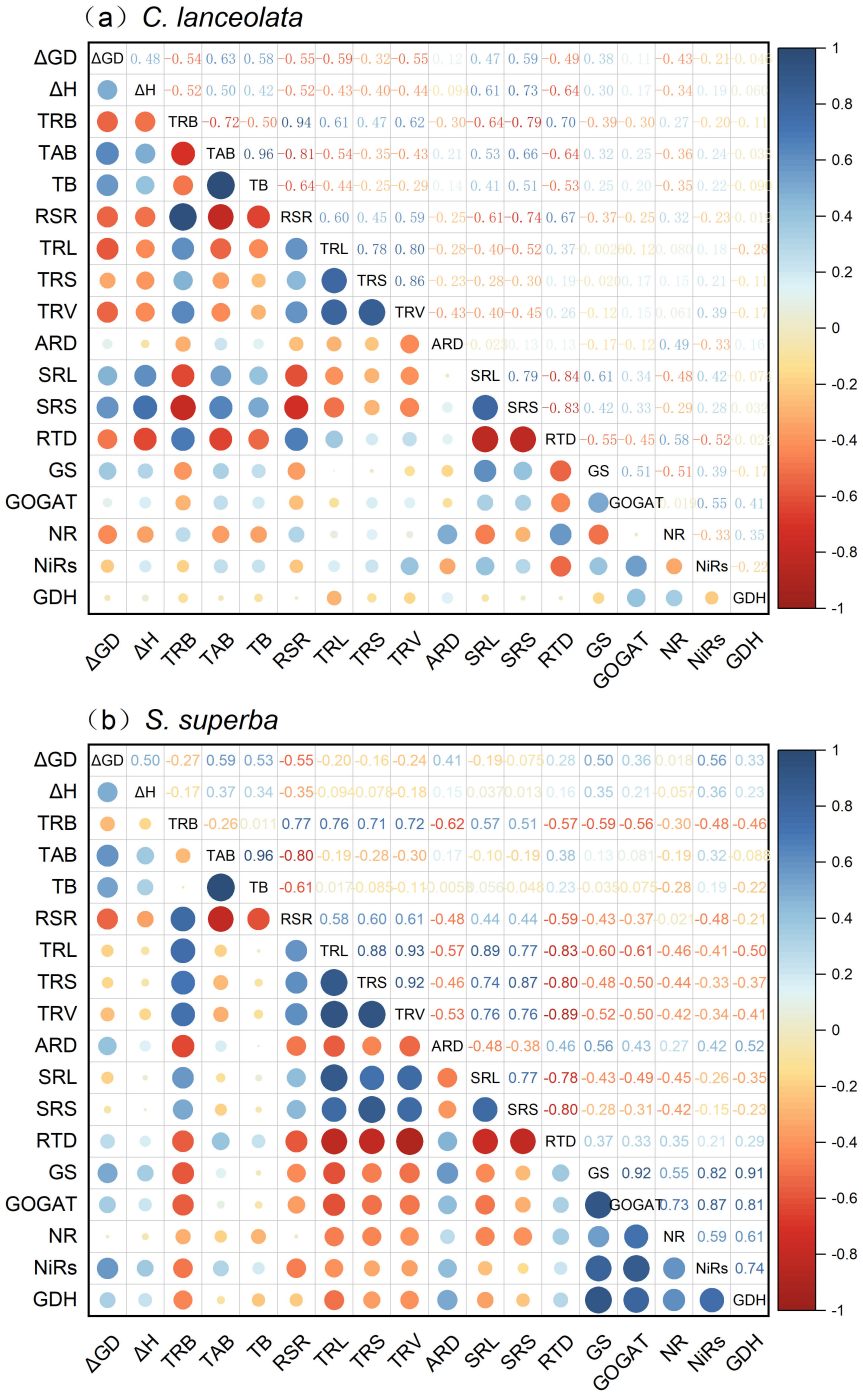
Correlation between growth, root morphology, and physiological traits of *C. lanceolata* **(a)** and *S. superba* **(b)** under N deficiency and NH_4_^+^-N to NO_3_^−^-N ratio. Blue indicates positive correlation coefficients, while red indicates negative correlation coefficients. The darker the color and the larger the bubble, the stronger the correlation; conversely, the lighter the color and the smaller the bubble, the weaker the correlation.

For *C. lanceolata*, the seedling growth traits (Δ*GD*, Δ*H*, TAB, and TB) show negative correlations with TRB SRS TRL TRS TRV RTD NR and GDH, and they show positive correlations with SRL SRS GS GOGAT. However, the enzyme activities (GOGAT, NR, NiRs and GDH) show weak to moderate correlations with seedling growth. The SRL and SRS of *C. lanceolat* were positively correlated with their GS, GOGAT, and NiRs and negatively correlated with NR, while the reverse was true for RTD. In addition, the correlations between the remaining root morphology and physiological traits were relatively weak (**Figure 6a**). For *S. superba*, seedling growth was relatively weakly correlated with morphological and physiological traits, but the correlation between morphology and physiology was strong. Among them, TRL, TRS, TRV, SRL, and SRS were negatively correlated with enzyme activities in the root system of *S. superba*, and the opposite was true for ARD and RTD (**Figure 6b**).

## Discussion

### The plasticity of root morphology under N deficiency and NH_4_^+^-N to NO_3_^−^-N ratio of *C. lanceolata* and *S. superba*

Plants often rely on the morphological plasticity of their roots to promote efficient nutrient uptake in response to scarcity or heterogeneous distribution of nutrients. Under adverse conditions, plants generally enhance their ability to acquire limited N resources by increasing root length, root surface area, and root volume, while simultaneously reducing average root diameter (Wang et al., 2013; Kou et al., 2015; Zhang et al., 2020). Previous studies have shown that *C. lanceolata* tends to grow more fine roots in NO_3_^–^N nutrient patches, whereas *S. superba* tends to grow more fine roots in NH_4_^+^-N nutrient patches (Yan et al., 2020a). Overall, our results align with previous studies. This may be related to the preference of *C. lanceolata* for absorbing NH_4_^+^-N. In environments with higher NO_3_^-^-N concentrations, the *C. lanceolata* adopts a N-seeking strategy involving a smaller root diameter in order to accelerate the uptake of limited nutrients and meet its own nutritional requirements. In contrast, *S. superba* exhibits relatively stronger plasticity responses under high NH_4_^+^-N concentrations. However, this study found that under conditions where the NH_4_^+^-N to NO_3_^-^-N ratio was 10:0, 2:8, and 0:10, respectively, N deficiency significantly inhibited root elongation in *C. lanceolata* while increasing its ARD. This resulted in roots exhibiting a ‘short and thick’ morphology, thereby diminishing nutrient uptake efficiency. This indicates that under the combined effects of N deficiency and an unfavorable NH_4_^+^-N to NO_3_^-^-N ratio, the root morphology response of *C. lanceolata* may exhibit divergent trends. This may be because plants experience rapid increases in stress-related hormones in their bodies when responding to adversity (Ding and Bai, 2021). Increased levels of phytohormones stimulate cell division and expansion, which can lead to an increase in average root diameter.

Furthermore, tree species constitute one of the most fundamental factors influencing N uptake. Over the course of long-term evolution, different plant species have developed distinct capacities for the absorption, assimilation and utilization of NH_4_^+^-N and NO_3_^-^-N. This has consequently shaped unique N form preferences and root foraging strategies (Zhou et al., 2022). This difference is clearly evident at the level of root morphology. For example, research has shown that SRL and SRS differ significantly between coniferous and deciduous species at the same root order level, with deciduous species generally exhibiting higher SRL and SRS values than conifers (Tang et al., 2015). In a short-term study on N additions in subtropical evergreen broadleaf species, *Castanopsis faberi* and *Castanopsis carlesii* have shown that the SRL and SRS of the lower-order roots (first to third order) of *C. faberi* increased, while the RTD was decreased. The SRL and SRS of the lower-order roots of *C. carlesii* decreased, while the RTD increased (Jia et al., 2019). This difference highlights the contrasting N foraging strategies of these two species. This study made one notable finding. The RTD of *S. superba* was higher than that of normal N supply at 0:10 NH_4_^+^-N to NO_3_^−^-N ratio and lower than that of normal N supply at the remaining four N ratios, suggesting that the two species adopt contrasting N foraging strategies influenced by specific NH_4_^+^-N to NO_3_^−^-N ratios. Roots with higher nutrient uptake capacity tend to be thinner, with higher SRL and N content, but with a shorter lifespan, while thicker, longer-lived roots have lower SRL and N content and exhibit more conservative resource use (Eissenstat, 1992). Therefore, *C. lanceolata* adopts a conservative N foraging strategy under N deficiency, reflected in reduced SRL and SRS and increased RTD. In contrast, *S. superba* follows a rapid nutrient uptake strategy under N-deficient conditions. In summary, the morphological plasticity of plant root systems is a key mechanism for responding to N availability and proportions of different morphological characteristics. However, this process is jointly regulated by the characteristics of the tree species, levels of N supply, and the NH_4_^+^-N to NO_3_^−^-N ratio.

### The plasticity of root physiology under N deficiency and NH_4_^+^-N to NO_3_^−^-N ratio of *C. lanceolata* and *S. superba*

The way plants regulate their N metabolism, depending on the form and level of N supply, is a complex process. It involves the synergistic action of multiple key enzymes and species-specific adaptive strategies. The results of our variance analysis in this study are in line with the conclusions of Goel and Singh, who found that different N supply levels and NH_4_^+^-N to NO_3_^–^N ratios have a significant impact on the activity of key N metabolism enzymes (Goel and Singh, 2015). When the supply of N is limited, the pathways of N metabolism in the plant are inevitably affected. Under N deficiency, plants often conserve resources by reducing energy-intensive metabolic processes in order to sustain essential life functions. This response pattern has been well documented in crops such as wheat and maize grains (Wang et al., 2003; Zhang et al., 2005). The activities of nitrate NR and GS decrease under N deficiency, exhibiting a sustained downward trend as the N concentration declines further. This study also observed similar patterns in forest trees. Under most NH_4_^+^-N to NO_3_^–^N ratios, the overall activity of GS, GOGAT, NR, NiRs, and GDH decreased in *S. superba* roots. The same trend was observed for GS, GOGAT, and NiRs in *C*. *lanceolata*. This indicates that plants generally reduce energy expenditure by lowering the activity of N-assimilation-related enzymes under N deficiency, thereby prioritizing limited resources for essential metabolic processes. NR is a key enzyme in the N metabolism process and also acts as both an inductive and rate-limiting enzyme in the N assimilation process, directly controlling the reduction of NO_3_^−^. This study observed increased NR activity in *C*. *lanceolata* under N deficiency, which is consistent with the adaptive strategy of plants to actively regulate N metabolism in response to N deficiency (Chen et al., 2016). This suggests that *C*. *lanceolata* can increase its ability to absorb limited NO_3_^−^-N by inducing nitrate reductase activity in its root system. This could be a way for the plant to adapt to environments with N deficiency. The assimilation of NH_4_^+^-N by plants mainly occurs through the GS/GOGAT cycle, synthesizing NH_4_^+^ into glutamine (Gln). The GS/GOGAT cycle is a critical process in N metabolism, and previous studies have shown that during the NH_4_^+^ assimilation process in crop growth periods, GS and GOGAT work together (Ma et al., 1996), and then GS and GOGAT generally exhibit the same trend. Different forms of N can increase the activity of GS in plants, with NH_4_^+^-N having a more pronounced effect, as it serves as a substrate for glutamine synthetase and can directly promote an increase in its activity. In this study, the patterns of GS and GOGAT in *C. lanceolata* and *S. superba* were generally consistent but did not align with the theory that high NH_4_^+^-N promotes the increase in GS. This suggests that GS activity is regulated by a variety of factors at different levels, such as carbon-nitrogen balance, energy status and transcription-translation processes, rather than solely by substrate concentration (Bao, 2013; Yang et al., 2015).

When the relative proportions of NH_4_^+^-N and NO_3_^−^-N in the soil change, plants adjust the activity of key enzymes involved in N metabolism to adapt to the changes in soil N. For example, when the proportion of NH_4_^+^-N in the soil increases, plants may upregulate the activities of GS and GOGAT to enhance their assimilation capacity for NH_4_^+^-N. Conversely, when the proportion of NO_3_^−^-N increases, plants may enhance the activities of NR and NiRs to promote the reduction and assimilation of NO_3_^−^-N. This evolutionary process has led to the formation of a preference for the absorption of different forms of N by plants over the long term. Studies have found that under mixed NH_4_^+^-N and NO_3_^−^-N treatments, the activity of NR in flue-cured tobacco first increases and then decreases with an increase in the proportion of nitrate N, while GS activity shows a continuous upward trend (Li et al., 2023). In this study, overall, the activities of GS, GOGAT, NR, and NiRs in *C. lanceolata* were relatively high at NH_4_^+^-N to NO_3_^−^-N ratios of 10:0 and 0:10, with the 5:5 ratio significantly enhancing the activities of GS and GOGAT in the roots. In summary, plant root systems demonstrate significant physiological plasticity. They respond to variations in different N supply levels and NH_4_^+^-N to NO_3_^−^-N ratios by dynamically regulating the activity of key enzymes involved in N metabolism.

### Effects of N deficiency and NH_4_^+^-N to NO_3_^−^-N ratio on seedlings growth of *C. lanceolata* and *S. superba*

The level of N supply and the ratio of NH_4_^+^-N to NO_3_^–^N significantly affect plant growth and development, as well as adaptability to environmental changes, by regulating plant-related morphogenesis and biomass allocation (Liu et al., 2016). Previous studies have shown that under N-deficient conditions, plants often exhibit characteristics such as stunted growth and reduced biomass. For instance, the root-to-weight ratio of *F. mandshurica* increased while total biomass decreased (Wu et al., 2004), and the height growth and dry matter accumulation of *Liriodendron chinense* were inhibited (Fan et al., 2009). This finding is consistent with the results of this study, which showed that N deficiency significantly inhibited the growth of seedling height and diameter in *C. lanceolata*, as well as the accumulation of both aboveground biomass and total biomass. However, the ratio of N forms can alter the effects of nitrogen deficiency stress. When the NH_4_^+^-N to NO_3_^-^-N ratio was 10:0, N deficiency actually promoted greater increases in height, aboveground biomass, and total biomass in *S. superba*. We hypothesize that although *S. superba* can efficiently utilize NO_3_^-^-N to maintain robust growth under normal N supply level, its reduction process incurs relatively high energy expenditure. Consequently, under N deficiency, *S. superba* may priorities the uptake of NH_4_^+^-N, which can be directly assimilated with lower energy consumption, thereby sustaining survival or growth under adverse conditions. Different ratios of NH_4_^+^-N to NO_3_^-^-N affect plant growth to varying extents, with these ratios varying widely between plants. The application of NH_4_^+^-N alone may induce potassium and calcium uptake disorders, or even ammonium toxicity (Yang et al., 2021), whilst the exclusive application of NO_3_^-^-N interferes with NH_4_^+^-N uptake (Xing and Ma, 2015). Consequently, mixed application typically optimizes metabolism and promotes growth (Wu et al., 2020; Sun et al., 2015; Wang et al., 2023). Under treatments with a single N source (either pure NH_4_^+^-N or pure NO_3_^-^-N), the growth of *C. lanceolata* showed no significant inhibition. However, the aboveground growth and biomass accumulation of *S. superba* were markedly restricted. This indicates that *S. superba* exhibits weaker adaptability to single nitrogen sources.

Changes in biomass allocation between above- and below-ground parts of plants are important strategies for plant adaptation to N deficiency. Root biomass is one of the important indicators to study the effect of nutrient addition on plant growth and development, and plant root biomass and its distribution reflect the adaptive self-regulation of different plants in response to different environments (Guo and Wang, 2021). It was found that root biomass also increased significantly in *C. lanceolata* at low N levels, but total biomass decreased (Cai et al., 2018; Wang et al., 2018). In this study, root biomass of *C. lanceolata* under N deficiency was higher than that under normal N supply at five NH_4_^+^-N to NO_3_^−^-N ratios, and root biomass of *S. superba* was higher than that under normal N supply at the remaining four NH_4_^+^-N to NO_3_^−^-N ratios, except for the NH_4_^+^-N to NO_3_^−^-N ratio of 2:8, indicating that under N deficiency, both tree species were able to obtain the nutrients required for growth by growing more roots. According to optimal allocation theory, when N supply is insufficient, plants will preferentially allocate carbon assimilates to organs with access to more limited resources by regulating the allocation of aboveground and aboveground biomass, leading to an increase in the plant’s root to crown ratio and an increase in root uptake rate through up-regulation of the high-affinity transporter system, thus increasing the efficiency of the plant’s use of limited N supply resources (Kobe et al., 2010).

In addition, the results of this study showed that the above-ground growth of *C. lanceolata* was strongly correlated with root morphology and physiological characteristics, whereas that of *S. superba* was relatively weak. According to the PCA analysis, *C. lanceolata* and *S. superba* showed significant differences in the principal component axes, especially in the PC1 axis. Thus, there was a clear difference in the root plasticity response between *C. lanceolata* and *S. superba*. Compared with normal N supply, the contributions of GS and GOGAT increased in the root system of *C. lanceolata* under N deficiency, indicating that it alleviated N deficiency by enhancing the N assimilation pathway (Xu et al., 2012). On the other hand, SRL and SRS of *S. superba* coordinated with each other and showed stronger contributions under N deficiency, indicating that it alleviated N stress mainly by changing morphological characteristics and maintaining a rapid uptake strategy to promote growth (Lynch, 2013). However, differences in the response of *C. lanceolata* and *S. superba* at different NH_4_^+^-N to NO_3_^−^-N ratios do not show a clear pattern. Tree species form ecological niche differentiation and resource utilization preferences during long-term evolutionary processes. When formulating differentiated fertilization and cultivation measures for future forest nitrogen management, the root response characteristics of individual tree species should be considered.

## Conclusion

N deficiency and different NH_4_^+^-N to NO_3_^−^-N ratios significantly affected the root morphological and physiological traits and seedling growth of *C. lanceolata* and *S. superba*. N deficiency promoted root growth and significantly increased the RSR of *C. lanceolata* and *S. superba* compared to normal N supply. Under N deficiency, TRB, TRL, TRS, TRV, and RTD increased, while ARD,SRL, and SRS decreased in *C. lanceolata* under the dominance of NO_3_^−^-N. In contrast, TRB, TRL, TRV, SRL, and SRS increased, while ARD and RTD decreased in *S. superba* under the dominance of NH_4_^+^-N. The key enzyme activities of root N metabolism in the two species exhibited different patterns in response to N supply levels. The growth of *C. lanceolata* and *S. superba* was inhibited under N deficiency, and the increase in NH_4_^+^-N concentration in the nutrient solution favored the TAB of the two species. Differences in root system plasticity in response to different N supply treatments in *C. lanceolata* and *S. superba*. In conclusion, the ability of *C. lanceolata* and *S. superba* to adapt to N deficiency and different NH_4_^+^-N to NO_3_^−^-N ratios through changes in root morphology, and physiological traits, as well as opposing N uptake strategies in *C. lanceolata* and *S. superba*, especially the N limitation was somewhat alleviated by the appropriate ratios of N forms.

## Data Availability

The original contributions presented in the study are included in the article/supplementary material. Further inquiries can be directed to the corresponding author.
